# Supplementary material for: Incidence and risk factors for heart failure in the ELSA-Brasil cohort
Source: PLoS One. 2025 Aug 12;20(8):e0329113. doi: 10.1371/journal.pone.0329113 (PMC12342299; doi:10.1371/journal.pone.0329113)

**FIGURES**

Statistical analyses were conducted using STATA software, version 16 (StataCorp, College Station, Texas, USA).

**Figure 1A: Median Age**
**Analyses:**
Quantitative variables vs. categorical variable: incident HF cases at the 2nd visit of the cohort.

Participants with baseline HF (n = 251) were excluded from this analysis.

**Graph 1A) Age vs. Incident HF cases at the 2nd visit:**

The age variable did not follow a normal distribution. The Mann-Whitney test was used instead of the t-test.


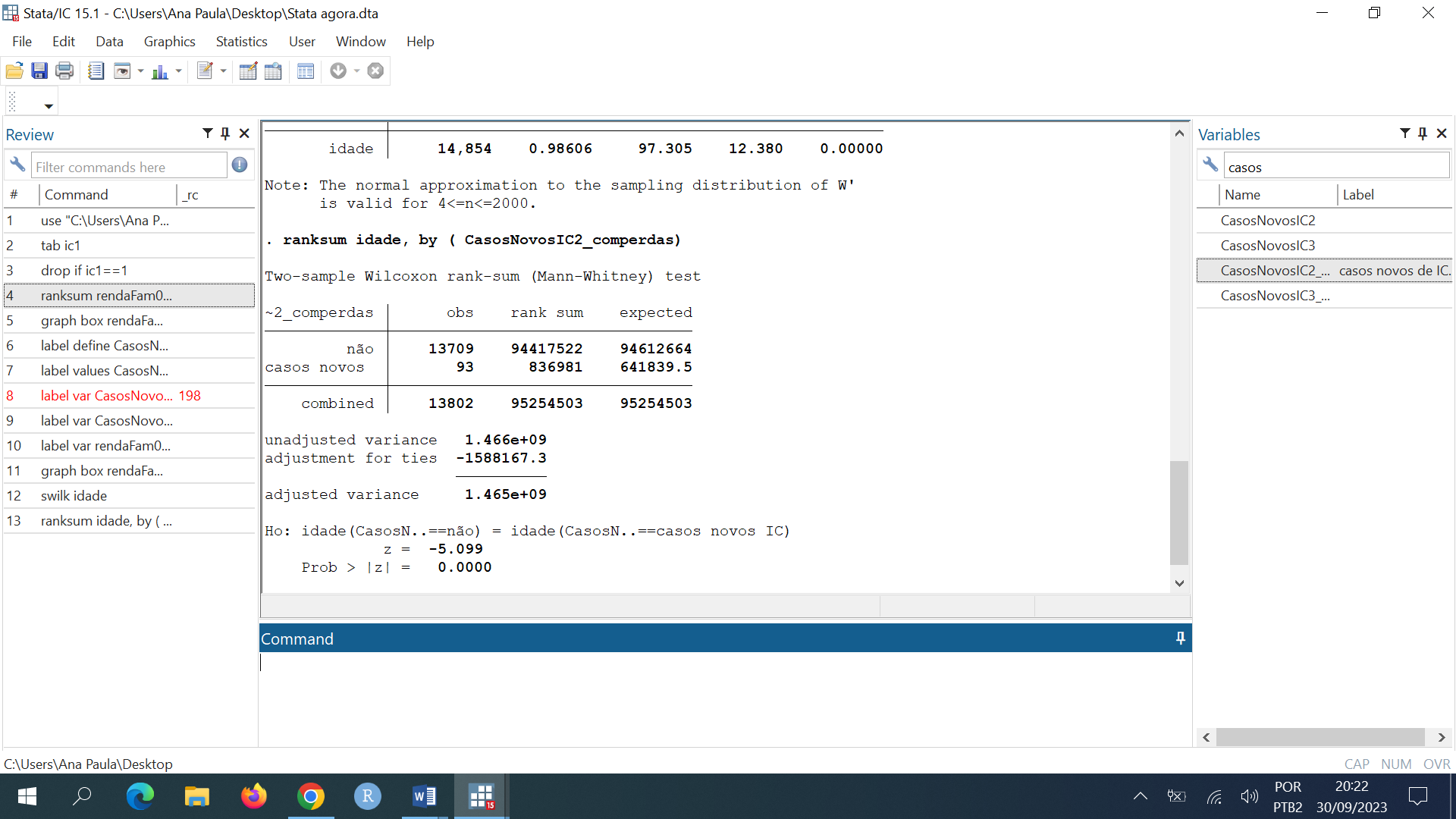


**Interpretation:**
There are differences in medians between the variable Age and incident Heart Failure cases in Wave 2, according to the Mann-Whitney test (p = 0.000). A statistically significant association was observed. Higher ages were associated with incident heart failure cases at the 2nd visit of the ELSA-Brasil cohort (Figure 1A).

**Figure 1A.** Box plot of age by incident heart failure status at visit 2 of the ELSA-Brasil cohort. Stata command used: graph box idade, over(Casos_novos).


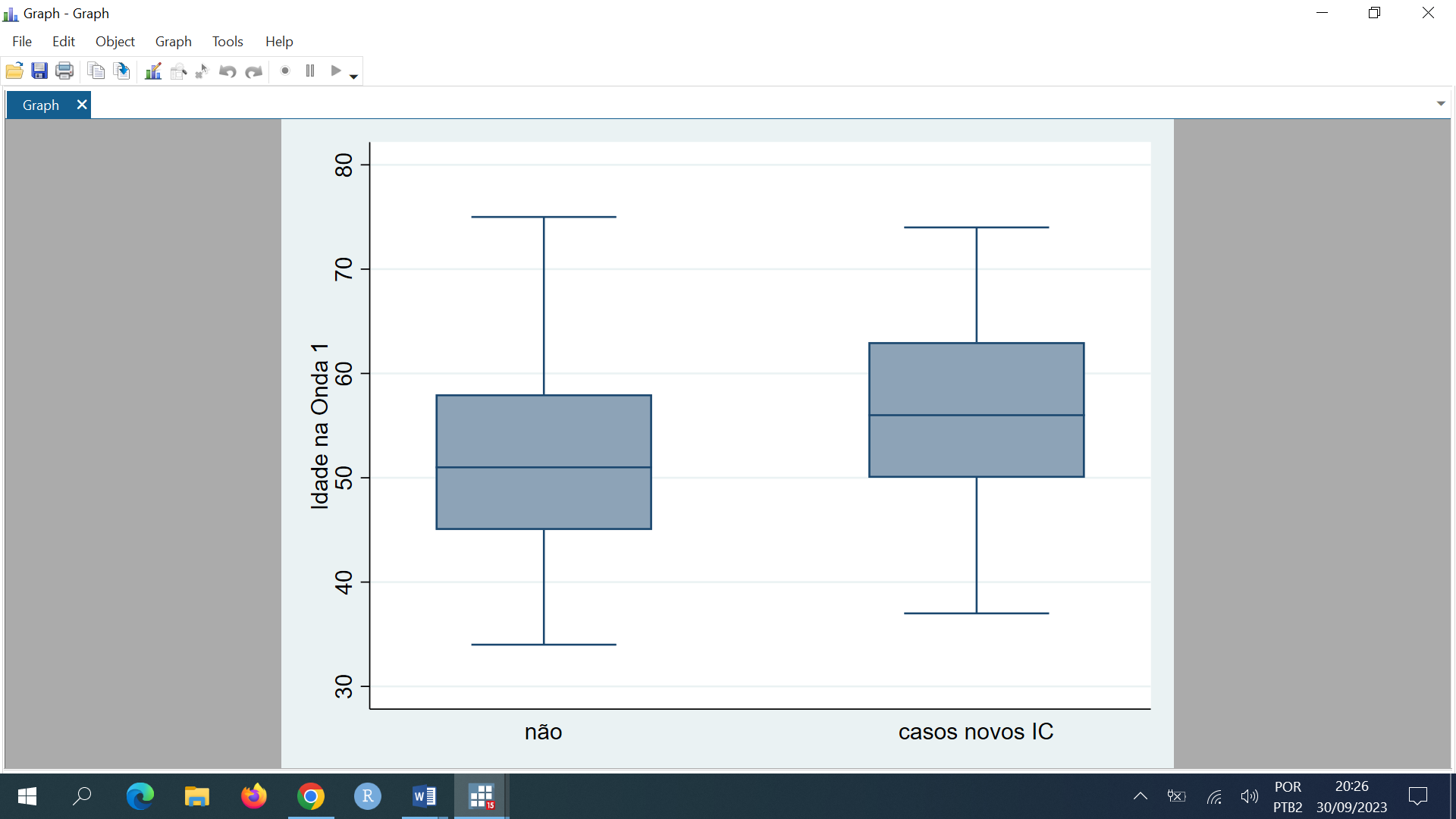


**Interpretation:** Box plot of baseline age from the ELSA-Brasil study, comparing participants who developed incident heart failure (HF) at visit 2 (n = 93) to those without HF. Median age was significantly higher among participants with incident HF compared to those who remained HF-free at visit 2 (p < 0.001; ELSA-Brasil, 2008–2014).

**Figure 1B: Waist Circumference**

Analyses:

Quantitative variable vs. categorical variable: incident HF cases at the 3rd cohort visit.

Participants with HF at baseline (n = 251) were excluded from this analysis.

**Figure 1B) Waist Circumference vs. Incident HF Cases at the 3rd Visit:**
Normality test for the waist circumference variable at Wave 1 = not normal.
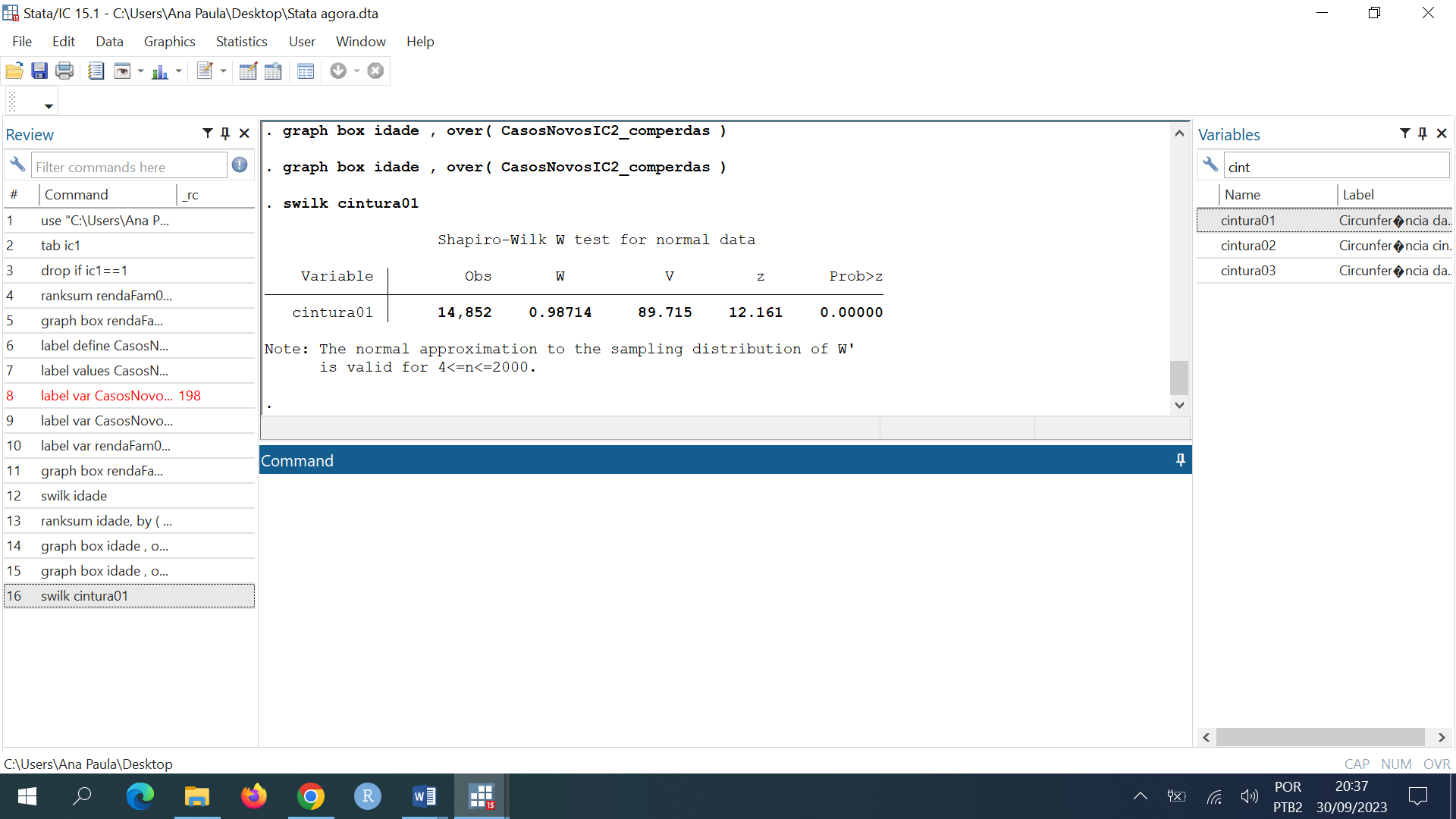


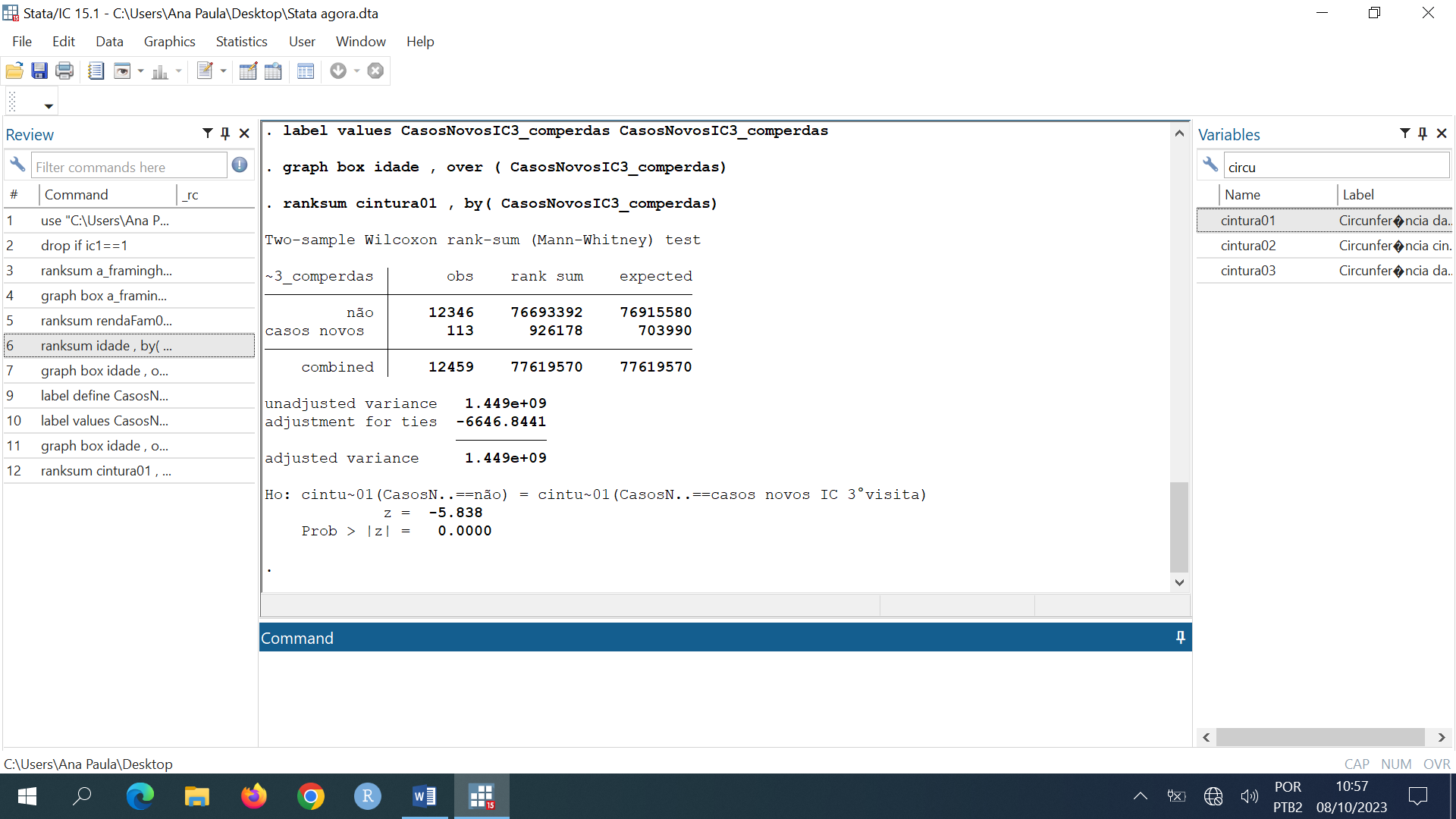


**Interpretation:** There was a significant difference in baseline waist circumference between participants who developed incident heart failure (HF) at the 3rd visit and those who did not, according to the Mann-Whitney test (p = 0.00). A statistically significant association was observed, indicating that higher waist circumference was associated with incident HF cases at the 3rd ELSA-Brasil visit (Figure 1B).

**Figure 1B.** Box plot of waist circumference by incident heart failure status at visit 3 of the ELSA-Brasil cohort. Stata command used: graph box cintura, over (Casos_novos)


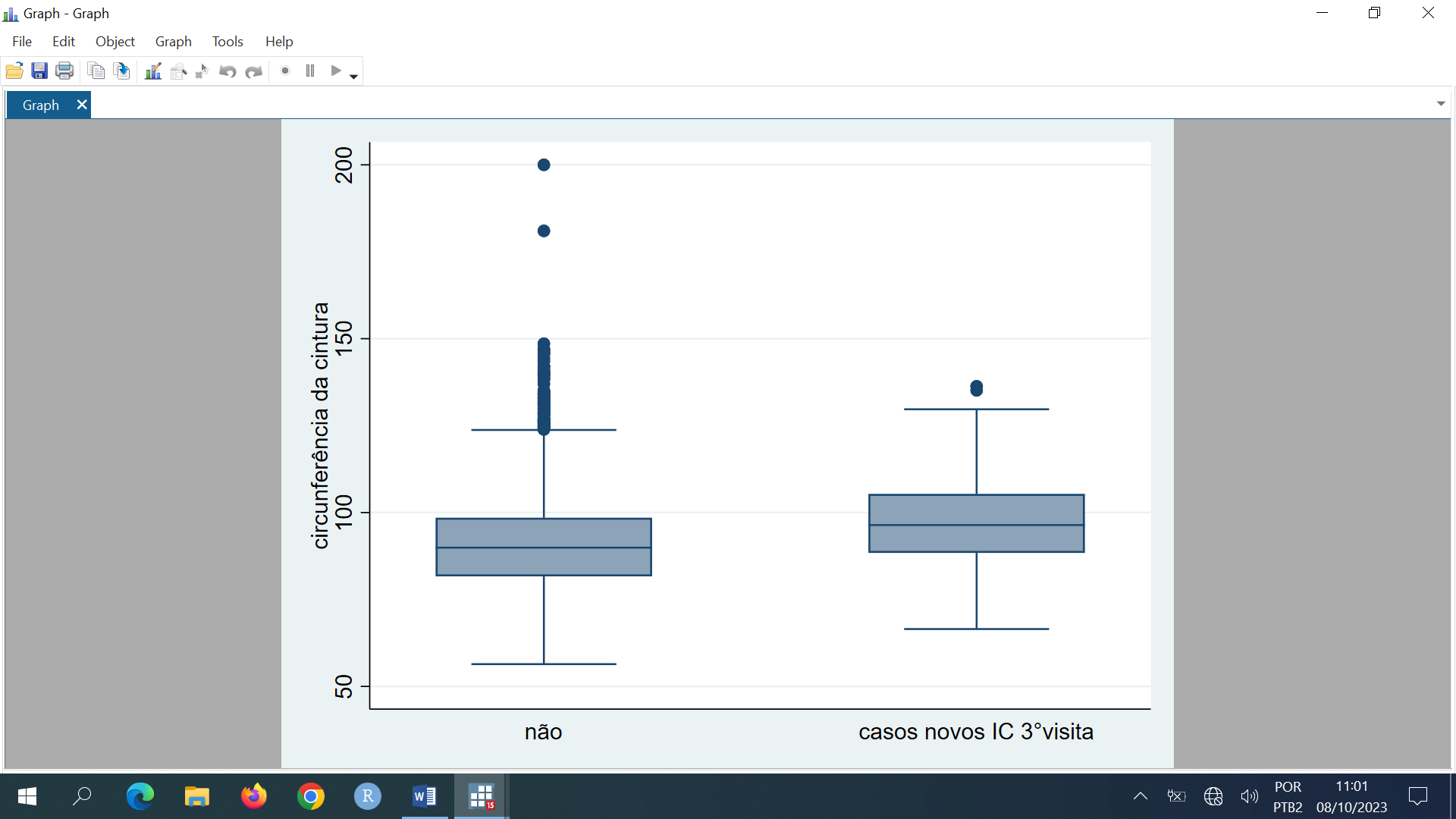


**Interpretation:** Box plot of baseline waist circumference by incident heart failure status at visit 3 (n = 113), comparing groups without HF ("No") and with new HF cases ("Incident HF"). Waist circumference (cm) was significantly higher in the group with incident heart failure at visit 3 of the ELSA-Brasil cohort (p = 0.00) (ELSA-Brasil, 2008–2018). Similarly, waist circumference was significantly greater among incident HF cases at visit 3 compared to participants without HF at baseline (p < 0.01).

**Variable age:**

**
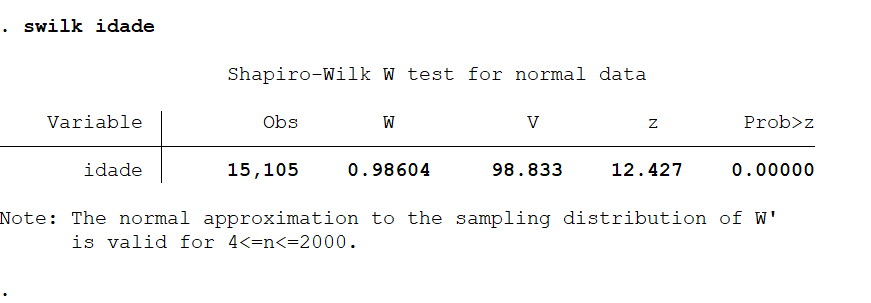
** The variable age does not follow a normal distribution according to the Shapiro-Wilk test.

This justifies the use of robust descriptive statistics for non-normal distributions, such as the median and interquartile range (Q1–Q3), and the use of non-parametric tests for comparisons, such as the Mann-Whitney test.

**Median of Age:**


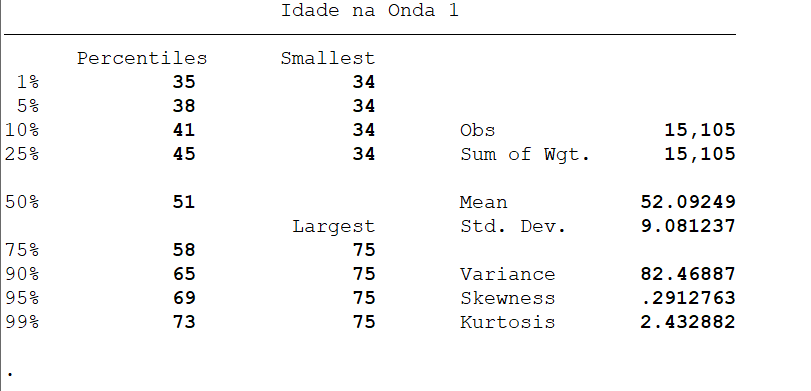


**Variable waist circumference**:


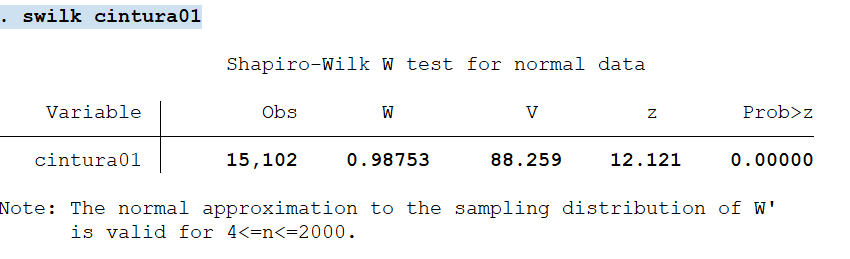


Justification for non-normality:

"The Shapiro-Wilk test indicated that the variable does not follow a normal distribution (p < 0.05). Therefore, non-parametric statistical methods and robust descriptive statistics such as medians and interquartile ranges were used for analysis

**Median of the variable waist circumference**


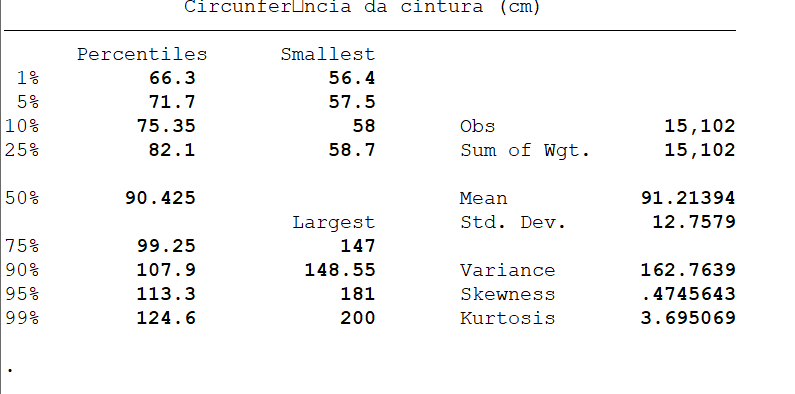

Supplement: S1 File — This file contains box plots and statistical analyses of baseline age and waist circumference comparing participants with and without incident heart failure at visits 2 and 3 of the ELSA-Brasil cohort. It includes tests of normality (Shapiro-Wilk), justification for using non-parametric tests (Mann-Whitney), and presentation of median values. (DOCX) [file pone.0329113.s001.docx]
